# Application of Artificial Intelligence in Pancreatic Cyst Management: A Systematic Review

**DOI:** 10.3390/cancers17152558

**Published:** 2025-08-02

**Authors:** Donghyun Lee, Fadel Jesry, John J. Maliekkal, Lewis Goulder, Benjamin Huntly, Andrew M. Smith, Yazan S. Khaled

**Affiliations:** 1 Leeds Institute of Medical Research, University of Leeds, Leeds LS2 9JT, UK; 2 The Pancreato-Biliary Unit, St. James’s University Teaching Hospital, Leeds LS9 7TF, UK

**Keywords:** pancreatic cyst, artificial intelligence, machine learning, benign, malignant, IPMN, MCN, diagnosis, prognosis

## Abstract

Background: Pancreatic cystic lesions (PCLs), including intraductal papillary mucinous neoplasms (IPMNs) and mucinous cystic neoplasms (MCNs), pose a diagnostic challenge due to their variable malignant potential. Current guidelines, such as Fukuoka and American Gastroenterological Association (AGA), have moderate predictive accuracy and may lead to overtreatment or missed malignancies. Artificial intelligence (AI), incorporating machine learning (ML) and deep learning (DL), offers the potential to improve risk stratification, diagnosis, and management of PCLs by integrating clinical, radiological, and molecular data. This is the first systematic review to evaluate the application, performance, and clinical utility of AI models in the diagnosis, classification, prognosis, and management of pancreatic cysts. Methods: A systematic review was conducted in accordance with PRISMA guidelines and registered on PROSPERO (CRD420251008593). Databases searched included PubMed, EMBASE, Scopus, and Cochrane Library up to March 2025. The inclusion criteria encompassed original studies employing AI, ML, or DL in human subjects with pancreatic cysts, evaluating diagnostic, classification, or prognostic outcomes. Data were extracted on the study design, imaging modality, model type, sample size, performance metrics (accuracy, sensitivity, specificity, and area under the curve (AUC)), and validation methods. Study quality and bias were assessed using the PROBAST and adherence to TRIPOD reporting guidelines. Results: From 847 records, 31 studies met the inclusion criteria. Most were retrospective observational (n = 27, 87%) and focused on preoperative diagnostic applications (n = 30, 97%), with only one addressing prognosis. Imaging modalities included Computed Tomography (CT) (48%), endoscopic ultrasound (EUS) (26%), and Magnetic Resonance Imaging (MRI) (9.7%). Neural networks, particularly convolutional neural networks (CNNs), were the most common AI models (n = 16), followed by logistic regression (n = 4) and support vector machines (n = 3). The median reported AUC across studies was 0.912, with 55% of models achieving AUC ≥ 0.80. The models outperformed clinicians or existing guidelines in 11 studies. IPMN stratification and subtype classification were common focuses, with CNN-based EUS models achieving accuracies of up to 99.6%. Only 10 studies (32%) performed external validation. The risk of bias was high in 93.5% of studies, and TRIPOD adherence averaged 48%. Conclusions: AI demonstrates strong potential in improving the diagnosis and risk stratification of pancreatic cysts, with several models outperforming current clinical guidelines and human readers. However, widespread clinical adoption is hindered by high risk of bias, lack of external validation, and limited interpretability of complex models. Future work should prioritise multicentre prospective studies, standardised model reporting, and development of interpretable, externally validated tools to support clinical integration.

## Simple Summary

Pancreatic cysts are common findings in the pancreas that can sometimes turn into cancer. However, it is often difficult for health care professionals to identify which cysts are potentially cancerous and which are not, leading to unnecessary surgeries or missed cancers. This study reviews how artificial intelligence (AI), including advanced computer programmes that learn from data, can help improve the diagnosis and management of pancreatic cysts. By analysing scans and patient information, AI models have shown promise in being more accurate than current guidelines or even doctors in identifying high-risk cysts. Although the early results are encouraging, many of these tools still need to be tested more thoroughly in real-world settings before they can be widely used in hospitals. This research highlights the potential of AI to make pancreatic cyst-care more accurate and personalised in the near future.

## 1. Introduction

Artificial intelligence (AI) is predicted to play an important role in modern medicine, offering new capabilities in data interpretation, pattern recognition, and decision support. By leveraging machine learning (ML) and deep learning (DL) algorithms, AI systems have the potential to enable the analysis of complex clinical, imaging, and molecular datasets to generate predictions with high accuracy and efficiency [1,2]. These tools are increasingly being adopted in diagnostic radiology, pathology, and oncology fields where precise decision-making is critical [3].

In pancreatic surgery, AI has shown promise in several domains, including the detection of pancreatic cancer, risk stratification, intraoperative navigation, and outcome prediction [4,5]. AI-based image analysis has been applied to improve diagnostic accuracy in pancreatic ductal adenocarcinoma (PDAC), automate segmentation, which refers to the process of identifying and outlining specific anatomical structures, and enhance radiological interpretation [6]. Over the past few years, there has been a growing area of interest in the application of AI to the management of pancreatic cystic lesions (PCLs), particularly intraductal papillary mucinous neoplasms (IPMNs) and other cystic neoplasms, which pose a diagnostic clinical challenge [7]. Pancreatic cysts are increasingly detected due to the widespread use of cross-sectional imaging [8]. While some pancreatic cystic neoplasms such as serous cystic neoplasm (SCN) are benign [9], IPMNs carry a risk of progression to invasive pancreatic cancer with risk of malignancy between 33 and 85% [10]. Differentiating high-risk cysts that require surgical resection from those amenable to surveillance remains a major diagnostic dilemma [11]. Existing guidelines, such as the Fukuoka and American Gastroenterological Association (AGA) criteria, rely on a combination of clinical and radiological features but are limited by moderate predictive accuracy [12,13]. A recent meta-analysis of studies that followed the Fukuoka and AGA Guidelines for predicting advanced neoplasia in pancreatic cyst neoplasm reported a sensitivity of 0.67 and 0.59, and specificity of 0.64 and 0.77, respectively [14]. Equally, the existing guidelines are associated with over treatment in some cases or missed malignancies in others [15]. Lekkerkerker et al. showed that 12% of pancreatic cysts with malignancy would have been missed under the AGA guidelines and fewer patients would undergo unnecessary surgery [16]. Other large observational studies suggested that there is no significant difference in the number of deaths between less-intensive and more-intensive surveillance of pancreatic cysts, highlighting the over screening and overtreatment of current guidelines [17,18].

AI offers an opportunity to enhance diagnostic precision by integrating radiological, clinical, and molecular data into models capable of identifying subtle patterns beyond human perception. In recent years, several AI-based models have been proposed to improve the preoperative assessment of pancreatic cysts including malignancy prediction, subtype classification, and risk stratification; yet these studies vary significantly in methodology, performance, and clinical relevance. For example, Tian et al. developed a convolutional neural network (CNN) model using MRI combined with clinical variables such as history of pancreatitis and diabetes to differentiate SCNs from mucinous cystic neoplasms (MCNs), achieving excellent performance with an area under the curve (AUC) of 0.97 [19]. Similarly, Chen et al. developed a logistic regression diagnostic model using CT images, gland texture-based features, cyst size, presence of calcifications, and central scarring [20]. This model also demonstrated strong diagnostic performance, with an AUC of 0.932. Both models aimed to distinguish SCNs from MCNs with high accuracy; however, they differed in the machine learning approach and imaging modality used. While the CNN model achieved slightly higher performance, its black-box nature limits interpretability. In contrast, the logistic regression model offers a transparent framework, allowing clinicians to understand the influence of individual variables on the model’s predictions, making it potentially more suitable for clinical integration.

While previous reviews have explored AI in pancreatic cancer and surgical applications broadly, there has been no systematic evaluation focused on the role of AI in the management of pancreatic cystic lesions. This systematic review is the first to identify and synthesise evidence from studies that apply AI, ML, or DL models to the diagnosis, classification, or prognosis of pancreatic cystic lesions. Here, we evaluate the performance, clinical relevance, and limitations of these models, and highlight research gaps to provide a framework for future studies in this emerging field.

## 2. Materials and Methods

This systematic review was conducted according to the Preferred Reporting Items for Systematic Reviews and Meta-Analyses (PRISMA) guidelines [21,22]. The protocol was prospectively registered on PROSPERO (registration number: CRD420251008593) in March 2025. The inclusion criteria for the study were the following: (i) original articles applying AI, ML, or DL to pancreatic cystic lesions (e.g., IPMN, MCN, and SCN), (ii) studies assessing diagnostic, classification, or prognostic performance, and (iii) human studies using radiological imaging (CT, MRI, and EUS), clinical data, or molecular features. Studies that focused solely on pancreatic cancer without reference to cystic lesions were excluded. Similarly, preclinical or animal studies were also excluded alongside editorials, case reports, conference abstracts, reviews, and articles not published in English.

Searches were conducted in PubMed, EMBASE, Scopus, and Cochrane Library using combinations of the following terms: pancreatic cyst OR pancreatic cystic neoplasm OR pancreas cystic lesions OR cystic lesions of the pancreas OR intraductal papillary mucinous neoplasm OR serous cystic neoplasm OR mucinous cystic neoplasm OR pseudopapillary tumour OR solid pseudopapillary tumour OR cystic neuroendocrine tumour AND artificial intelligence [All Fields] OR artificial intelligence [MeSH Terms] OR machine learning [All Fields] OR deep learning [All Fields]. The final search was performed in March 2025.

### 2.1. Study Selection

Title and abstract screening were conducted by two independent reviewers, with the final selection validated by the senior author (PB consultant surgeon). Full-text screening was performed using pre-defined inclusion/exclusion criteria. Disagreements were resolved through consensus or discussion with the senior author.

### 2.2. Data Extraction

The following data were extracted for each included study: title, year of publication, study design, number of patients, imaging modality used (CT, MRI, and EUS), and type of AI/ML model used. For each study, the clinical, radiological, and molecular parameters were collected against the AI model. Outcome endpoints for each study included sensitivity, specificity, and AUC for diagnosis, grading, and malignancy prediction when available. Prognostic or complication-related outcomes were also recorded when available.

## 3. Results

**Figure 1:**
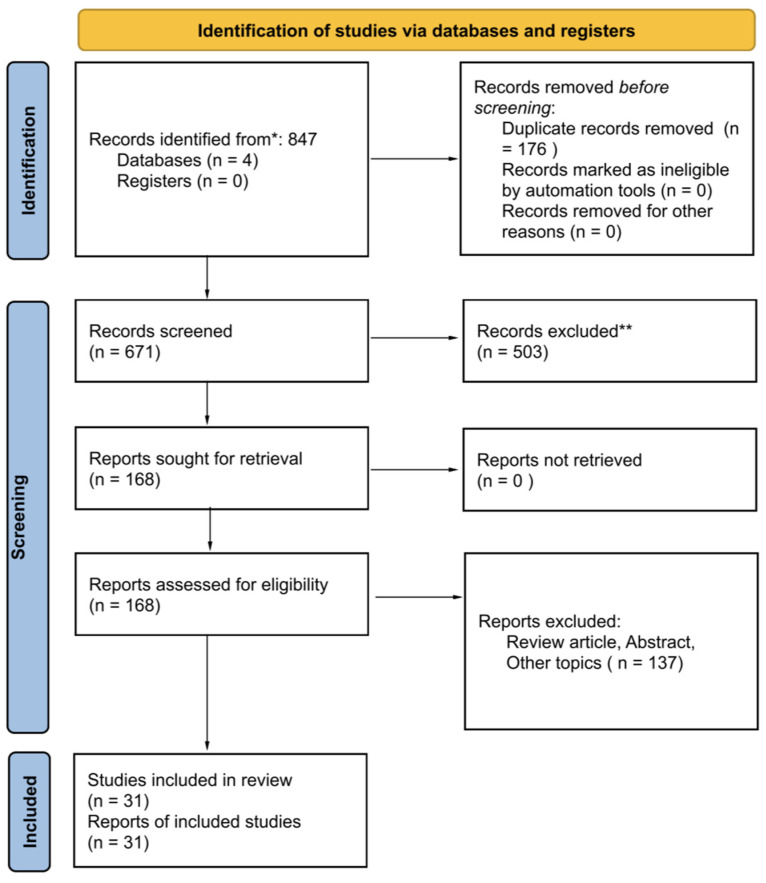
PRISMA flow diagram illustrating the study selection process for this systematic review. The flow outlines the number of records identified through database searches (*), the number of duplicates removed, studies screened, full-text articles assessed for eligibility, and studies included in the final review. There were 503 studies excluded (**), mostly because the studies did not address the use of artificial intelligence or did not focus on pancreatic cysts specifically. * represent the total number of record identified. ** represents the studies excluded in the study.

A total of 847 articles were identified. After duplicate removal and title/abstract screening, 168 articles were reviewed in full. A total of 31 studies met all the inclusion criteria and were included in the final analysis. No additional studies were identified via cross-referencing. The full selection process is summarised in the PRISMA flow diagram (Figure 1).

**Figure 2:**
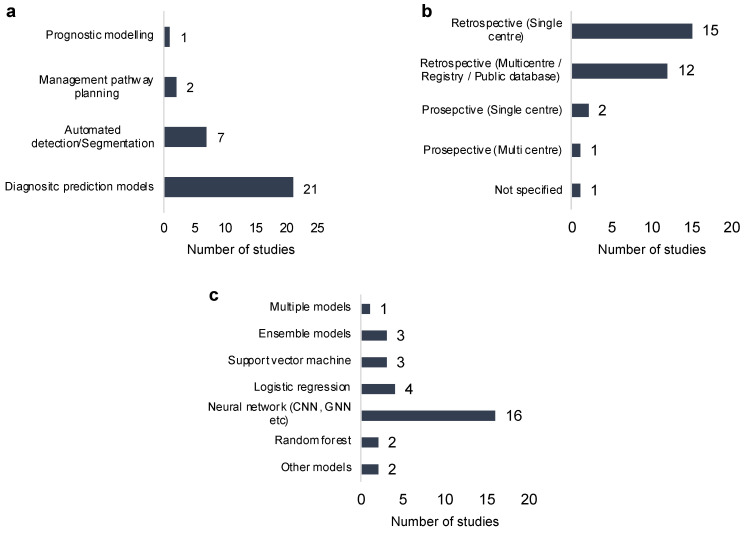
(**a**) Sub-domains of included studies in the review, with majority of studies on diagnostic prediction models; (**b**) characteristics of included studies according to design; and (**c**) machine learning models employed/developed.

The included studies were predominantly retrospective observational (n = 27) in design. One study was a retrospective internal pilot study, while only three of the studies found were prospective (Figure 2a). No randomised controlled trials were found. Only one study investigated prognosis, while the remaining thirty focused on preoperative diagnostic tasks. Most studies were published between 2019 and 2024. The only prognosis study reported its length of follow-up to be 5 years, with a median patient age of 66.

Most of the studies used imaging as a main parameter to reach the end point. These included CT scans, which accounted for 48%, and EUS and MRI, which accounted for 26% and 9.7%, respectively. Two studies used all three imaging modalities to reach their end point. Conversely, three studies did not use imaging modalities, but other parameters such as clinical symptoms, histological results, or other diagnostic biomarkers. Additionally, 11 studies employed clinical, demographic, or biological parameters such as carbohydrate antigen 19-9 (CA19-9) to further narrow down the end point. The number of patients used for each study ranged from 35 to 18,769.

We broadly categorised the included studies into two major domains: preoperative diagnostic applications of AI and postoperative applications. Of the 31 studies included in this review, 30 focused on preoperative diagnostic tasks (Figure 2b). These preoperative studies were further subcategorised into three functional areas: diagnostic prediction, where AI models were developed to predict the malignancy of pancreatic cysts or classify cysts into specific histological subtypes; automated detection and segmentation, where machine learning algorithms were used to segment suspected pancreatic cysts and identify lesions, thereby reducing the number of missed cases; and management pathway planning, where AI was utilised to aid clinical decision-making regarding surveillance, surgical intervention, or discharge. Of the 30 preoperative studies, 70% focused on diagnostic prediction, 23.3% on automated detection and segmentation, and 6.7% on management decision support.

While some studies from domain 1 (diagnostic prediction models) specifically focused on intraductal papillary mucinous neoplasms (IPMNs), others examined a broader range of pancreatic cystic lesions. Despite the variation in scope, the findings were largely consistent, supporting the potential role of AI in aiding the diagnosis and management of pancreatic cysts and their malignant potential.

Only one study addressed the prognosis of pancreatic cysts. Its endpoint was the prediction of long-term outcomes post-surgery, specifically 5-year disease-specific survival. It developed an AI model using clinicopathological variables such as age, tumour stage, and nodal involvement. Although based on postoperative data, the model demonstrates the potential of AI to support postoperative risk stratification, particularly in guiding adjuvant therapy and tailoring follow-up strategies. Most studies were published in the United States (n = 10), followed by China (n = 9) and South Korea (n = 3).

### 3.1. Machine Learning Methods

The most common machine learning model adopted by studies was a neural network (n = 16), specifically convolutional neural networks. Other ML models included logistic regression (n = 4), support vector machine (n = 3), ensemble models (n = 3), and random forest (n = 2). One model utilised multiple models to compare the performance of each individual model (Figure 2c). With regard to validation method, most studies adopted k-fold cross validation (n = 14), followed by simple train-test split (n = 6) and hold-out validation (n = 1). In total, 32.3% of the included studies performed external validation using an independent cohort.

### 3.2. Diagnosis and Subtyping

Many of the models aimed to stratify the malignant potential of pancreatic cysts, either directly by distinguishing benign from malignant lesions, or indirectly by classifying cysts into histological subtypes. For example, differentiating between serous cystadenomas (typically benign) and mucinous cystic neoplasms (with malignant potential) indirectly informs malignancy risk. Similarly, models that grade IPMNs into low-risk and high-risk categories also serve as an indirect method of malignancy prediction.

Benign vs. malignancy classification was addressed by four studies (Appendix A Table A1). Of these, three utilised CT imaging, while one employed EUS as the primary modality. Two studies further incorporated clinical parameters such as sex, age, history of pancreatitis, and serum biomarkers including CA19-9 and carcinoembryonic antigen (CEA). Radiological features commonly assessed included lesion location, size, and main pancreatic duct dilation. Sample sizes across these studies ranged from 27 to 388 patients. Reported performance metrics included accuracies between 84% and 99%, and AUC values ranging from 0.91 to 0.948. The machine learning approaches used were primarily convolutional neural networks (CNNs, n = 2), followed by logistic regression (n = 1), and an ensemble model (n = 1).

Subtypes classification was explored in nine studies, which developed and validated machine learning models aimed at classifying pancreatic cysts into specific subtypes or distinguishing one cyst type from another (Appendix A Table A2). The majority utilised CT imaging (n = 7), with others employing MRI (n = 1) and EUS (n = 1). In addition to radiological assessments, several studies incorporated radiomic features such as cyst size, shape, presence of a central scar, calcifications, and texture characteristics. Clinical parameters—including age, sex, diabetes, and jaundice—were also integrated in some models. The classification tasks spanned mucinous versus non-mucinous differentiation, as well as subtype identification including serous cystic neoplasm (SCN), mucinous cystic neoplasm (MCN), intraductal papillary mucinous neoplasm (IPMN), solid pseudopapillary neoplasm (SPN) and pancreatic neuroendocrine tumour (PNET). Machine learning approaches were diverse, including convolutional neural networks (CNNs, n = 3), random forests (n = 2), logistic regression (n = 2), support vector machines (SVM, n = 1), and ensemble methods (n = 1). Sample sizes varied from 28 to 314 patients. Performance metrics were robust, with reported accuracies between 72% and 98.5%, and AUC values ranging from 0.72 to 1.00.

IPMN stratification, addressed in eight studies, specifically targeted the classification and risk stratification of intraductal papillary mucinous neoplasms (IPMNs), focusing on differentiating low-grade, high-grade, and invasive subtypes (Appendix A Table A3). Imaging modalities included EUS (n = 4), MRI (n = 2), and multimodality approaches combining CT, MRI, and EUS (n = 1), while one study did not specify the imaging technique. These models incorporated a broad array of features including demographic variables (age and sex), clinical history, and biochemical markers such as CA19-9, CEA, and amylase. Radiological and radiomic parameters commonly assessed included cyst size, main pancreatic duct (MPD) diameter, presence of mural nodules, ductal dilatation, and papillary epithelial thickness. Among the EUS-based studies, one incorporated probe electrospray ionisation mass spectrometry (PESI-MS) analysis of cyst fluid in addition to EUS imaging, achieving an accuracy of 71.4% in distinguishing low-grade, high-grade, and invasive IPMN. Another EUS-based study utilised needle-based confocal laser endomicroscopy (nCLE) to evaluate microscopic epithelial features, reporting accuracies between 82.9% and 85.7% in stratifying low- versus high-grade lesions. A variety of machine learning models were employed across the studies, including convolutional neural networks (CNNs, n = 3), support vector machines (SVM, n = 2), logistic regression (n = 1), vision transformer neural networks (ViT, n = 1), and mixed-model approaches (n = 1). Sample sizes ranged widely, from 35 to 3708 patients. Reported model performance was generally strong, with accuracies ranging from 70% to 99.6%, and AUC values between 0.725 and 0.98.

Segmentation and automated identification of pancreatic cystic lesions (PCLs) were addressed in seven studies, utilising a variety of imaging modalities and machine learning techniques (Appendix A Table A4). Most employed CT imaging (n = 5), while others used EUS (n = 2). Manual segmentation of cyst regions or regions of interest (ROI) was a common step in the training process across several studies. The models aimed to either detect the presence of PCLs or segment them from the surrounding pancreatic tissue. Deep learning approaches were predominantly used, including convolutional neural networks (CNNs, n = 4), U-Net architectures (n = 2), and vision transformers (ViT, n = 1). One study employed a natural language processing (NLP) framework to identify cyst-related terminology in radiology reports, achieving high specificity of 0.99 but low sensitivity of 0.33. Reported model performance across the other studies was generally strong, with accuracies ranging from 82.9% to 97.2%, AUC values between 0.87 and 0.98, and sensitivities up to 93.1%. Sample sizes ranged from 111 to 18,769 patients.

### 3.3. Management Support Models

Two studies developed decision support models aimed at guiding the clinical management of pancreatic cystic lesions by categorising patients into treatment pathways such as surgery, active monitoring, or discharge (Appendix A Table A5). One study used data from 850 patients, incorporating clinical variables (age, gender, race, and symptoms), cyst characteristics (size and number), and cystic fluid molecular markers (CFMM) including vascular endothelial growth factor (VEGF), CEA, and Von Hippel–Lindau (VHL) mutations [23]. Although the imaging modality was not specified, the study employed an ensemble machine learning model, achieving classification accuracies of 93% for discharge, 84% for monitoring, and 83% for surgical referral. The second study used a supervised machine learning approach (model type not specified) and included 862 patients with integrated clinical, molecular, and multimodal imaging data (CT, MRI, and EUS) [24]. It reported a surgical accuracy of 91%, discharge accuracy of 60%, and an overall accuracy of 69%. These models underscore the potential of AI-driven tools to support personalised management decisions in patients with pancreatic cysts.

### 3.4. Prognostic Models

One study focused on prognostic modelling to predict long-term outcomes following surgical treatment for invasive IPMN (Appendix A Table A5) [25]. Using a cohort of 440 patients, the model incorporated demographic variables (age and gender), tumour characteristics (size, location, and histological grade), treatment details (type of surgery, radiotherapy, and chemotherapy), year of diagnosis, and TNM staging. The aim was to predict five-year disease-specific survival using both an artificial neural network (ANN) and a logistic regression model. The models achieved comparable performance, with reported accuracies between 81% and 82%, and precision scores ranging from 0.83 to 0.863.5. 

**Figure 3:**
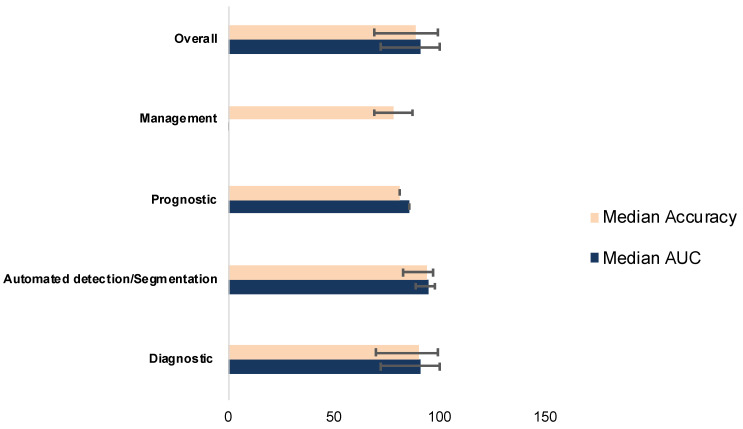
Range and median of AUC values across studies grouped by diagnostic domain (e.g., diagnosis, segmentation, risk stratification, management, and prognosis). Each bar displays the minimum and maximum AUC values reported in the studies within that domain, with the central marker indicating the median AUC.

The performance of models was evaluated via AUC, sensitivity, specificity, and accuracy. The range and percentage of studies with values >80% were as follows: AUROC (0.725–1, 54.8% (n = 17)) and accuracy (69–99.6, 58.1% (n = 18)). Median AUROC and accuracy values of each domain are depicted in Figure 3.

Seventeen studies compared machine learning models to radiologists, surgeons, clinician diagnosis, existing guidelines, or traditional logistic regression model. Seven studies compared the models to clinicians including junior radiologists, senior radiologists, surgeons, or other clinicians in the diagnosis of cyst subtypes and identification of pancreatic cysts [26,27,28,29,30,31,32]. Of these, four studies showed overall better performance in the machine learning model compared to clinicians, while three studies performed similarly [26,27,29,32]. Despite the better performance, one study argues that there was no statistically significant difference due to the small sample size [27]. Eight studies from similar domains as the above seven studies compared their models to existing guidelines such as Fukuoka, European, or American Gastroenterological Association (AGA) [23,24,33,34,35,36,37,38]. Seven of them showed better accuracy and sensitivity compared to the guidelines [23,33,34,35,36,37,38]. Another study claims that their model can support with management planning, correctly changing the plan by 25% [23]. Three studies compared the models’ performance to traditional logistic regression, with two studies arguing that the models were comparable to LR [25,39]. Only one study demonstrated that their model performed better than LR generally [29]. A summary of these findings can be found in the conclusion column of Appendix A Table A1, Table A2, Table A3, Table A4 and Table A5.

### 3.5. Risk of Bias Assessment

**Figure 4:**
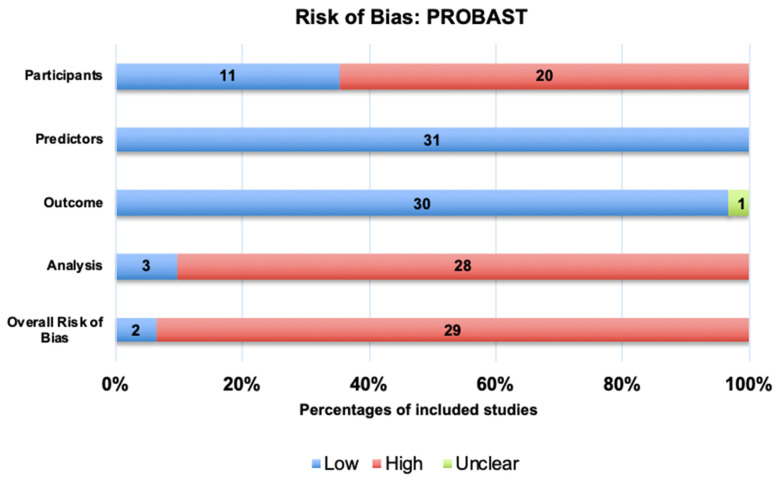
Risk of bias of included studies using Prediction Model Risk of bias assessment tool (PROBAST).

Study quality was assessed using the Prediction Model Risk of bias assessment tool (PROBAST) [40]. Domains included participants, predictors, outcomes, analysis, and overall. Studies were grouped by primary objective: (1) preoperative diagnosis and (2) prognosis prediction, and the results are shown in Figure 4.

**Figure 5:**
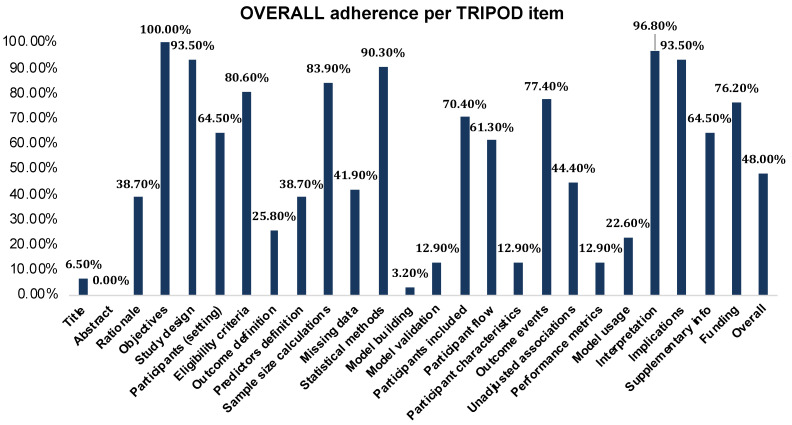
Reporting adherence of included studies to transparent reporting of a multivariable prediction model for individual prognosis or diagnosis (TRIPOD) tool.

The overall adherence to the transparent reporting of a multivariable prediction model of individual prognosis or diagnosis (TRIPOD) checklist across included studies was 48.0%, with adherence rates falling below 50% in 14 of the 25 evaluated reporting domains (Figure 5). Adherence exceeded 90% for reporting study objectives, study design, statistical methods, interpretation of findings, and clinical implications. Conversely, the lowest adherence rates (below 15%) were observed for title reporting, abstract reporting, model building strategies, model validation processes, participant characteristics, and reporting of performance metrics. Furthermore, fewer than 30% of studies adequately defined outcome measures and model usage. Reporting on participant flow and inclusion achieved rates of 61.3% and 70.4%, respectively, while funding disclosures were present in 76.2% of studies [41].

### 3.6. High Quality Studies

Two studies were assessed as having low risk of bias across all PROBAST domains. Sijia et al. developed a logistic regression model combined with radiomic features and clinical biomarkers to classify the histological grade of branch-duct IPMNs. The study included a multicentre cohort and achieved strong performance, with an AUC of 0.903 in the training cohort and 0.884–0.876 in two external validation sets. The authors clearly defined the study population, used consistent predictor definitions, and incorporated an interpretable nomogram to guide clinical decision-making. Importantly, they performed external validation and reported calibration, enhancing real-world applicability of their model [42]. Jae Seung et al. conducted a large, multicentre study involving 3708 patients, using data from CT, MRI, and EUS imaging along with clinical features to classify IPMN risk. The authors compared multiple models, including ensemble machine learning methods and logistic regression, and reported a mean AUC of 0.725 across modalities [39].

In addition to the two studies assessed as low risk of bias by PROBAST, we identified eight other studies that demonstrated strong clinical performance, external validation, and comparability or superiority to existing clinical guidelines or human readers. These studies collectively addressed a range of diagnostic and management tasks including malignancy prediction, subtype grading, segmentation, and clinical decision support. A summary of these high-quality, externally validated studies is presented below in Table 1. This comparative synthesis highlights each study’s AI model, validation status, clinical focus, key performance metrics, comparative advantage over current standards, and known limitations. These findings underscore the growing robustness and translational potential of machine learning applications in pancreatic cyst management.

## 4. Discussion

This systematic review evaluated 31 studies published between 2019 and 2024, providing a comprehensive overview of the current use of artificial intelligence in the diagnosis, management, and prognosis of pancreatic cystic lesions. While machine learning has been more widely applied in pancreatic cancer and pancreatitis, dedicated studies focusing on pancreatic cysts remain limited. Most of the included studies were retrospective and conducted at single centres. To our knowledge, this is the first systematic review to specifically investigate the role of machine learning in the context of pancreatic cysts. Most studies (n = 21) aimed to predict the malignancy of pancreatic cysts or classify them into histological subtypes, indirectly informing malignancy risk. Reported model performance was generally strong, with a median area under the receiver operating characteristic curve (AUROC) of 0.912 across studies. Of the studies reporting AUC, 54.8% achieved a value of ≥0.80, indicating high diagnostic discrimination. Several studies compared their AI models with existing clinical guidelines or human performance. Of clinical interest, seven models outperformed established guidelines such as Fukuoka or AGA [26,27,28,29,30,31,32]. Four studies demonstrated that their models outperformed clinicians in diagnostic accuracy, [26,27,29,32] and one showed superior performance to traditional logistic regression [29]. These findings support the potential of AI to enhance diagnostic decision-making beyond current standard approaches.

External validation, a critical component in evaluating the generalisability of machine learning models, is used to assess the feasibility of applying these models in real-world clinical settings. It typically involves testing a developed model on an entirely independent dataset that was not used during model training or internal validation phases, thereby providing insight into the model’s robustness and applicability across diverse patient populations [44]. Despite the significance of external validation, it was performed in 10 studies, representing just 32.3% of those reviewed.

### 4.1. Clinical Applicability and Integration

In this review, we focused on a range of pancreatic cystic lesions, including SCNs, MCNs, and IPMNs. Pancreatic cysts are most often detected incidentally with rate of detection being 8%, as they are frequently asymptomatic [45]. Importantly, not all cysts require surgical resection; however, IPMNs and MCNs carry a risk of malignant transformation and may warrant operative management [45,46]. Currently, the primary imaging modalities used in the evaluation of pancreatic cysts include CT, MRI, and endoscopic ultrasound (EUS) [47]. Despite their widespread use, several studies have highlighted limitations in their diagnostic accuracy [48,49]. While endoscopic ultrasound-guided Fine-Needle Aspiration (EUS-FNA) enables both morphological and cytological analysis of pancreatic lesions, it is associated with certain limitations and potential complications including pancreatitis and abdominal pain, with an overall rate of 2.9% and 2.2%, respectively [50,51,52]. Kirsten et al. reported that although EUS-FNA has a relatively low false-positive rate, its false-negative rate, particularly in the evaluation of solid and cystic pancreatic lesions, is notably higher, with a rate of 25% (95% CI, 16–36%), potentially delaying the diagnosis of malignancy [53]. Although various clinical guidelines exist, the management of pancreatic cysts must be individualised, considering a patient’s overall health status, malignancy risk, and personal preferences [49].

While AI’s potential to augment diagnostic accuracy is evident, its integration into clinical workflows remains limited. Few studies explored how AI tools might change management decisions or improve patient outcomes. Only two studies proposed decision-support models capable of guiding surveillance versus surgical intervention. One study demonstrated that an AI model integrating radiological, clinical, and cyst fluid biomarkers improved surgical triage accuracy and reduced unnecessary resections by 59% and improved the rate of correct surgeries by 7.5% [23]. Such examples highlight the promise of AI to individualise management and enhance decision-making, but their adoption requires regulatory approval, interpretability, and clinician trust. In daily clinical practice, free and open-source tools such as PyRadiomics and 3D Slicer exist to facilitate semi-automated segmentation and radiomic feature extraction from medical imaging [54,55]. However, these tools are not standalone diagnostic systems but rather function as technical components to support model development. To date, no machine learning models specific to pancreatic cysts are commercially available or approved for routine clinical use. Paid platforms remain in development and are typically confined to research settings. Importantly, there are no FDA- or CE-approved AI tools specifically for pancreatic cyst diagnosis currently in routine clinical use. As such, the optimal model developmental strategy for this purpose remains unclear. While this review focuses on pancreatic pathology, the clinical application of AI in diagnostic imaging and risk stratification has had a significant impact across other medical specialties. For example, AI systems have demonstrated performance surpassing that of human experts in breast cancer prediction. McKinney et al. curated a large representative mammographic imaging dataset from the UK and USA and showed that their AI model allowed an absolute reduction in false positives by 5.7% (USA) and 1.2% (UK), and in false negatives by 9.4% (USA) and 2.7% (UK) [56]. End-to-end lung cancer screening with three-dimensional deep learning on low-dose chest CT showed similar impact with absolute reductions of 11% in false positives and 5% in false negatives [57].

Deep learning models such as CNNs are powerful learning techniques widely used for image-based tasks due to their exceptional pattern recognition capabilities. Dominik et al.’s study involved EUS images combined with clinical parameters, achieving remarkable accuracy (99.6%), sensitivity (100%), and specificity (99.7%) [38]. This model notably outperformed standard clinical guidelines (AGA, Fukuoka, ACG, and European) [12,13,47,58]. However, the black-box nature of CNNs limited the interpretability of these outcomes. Few studies incorporated explainability methods, such as attention maps or feature attribution techniques, which are essential for clinician acceptance and safe implementation.

Artificial neural networks (ANNs) are a class of deep learning models, which can analyse non-linear relationships between input features and outcomes. Thereby, it is possible to analyse high-dimensional data and predict outcomes like malignancy risk or long-term survival. Least absolute shrinkage and selection operator (LASSO) is a type of linear regression that is capable of selecting only the most important variables, shrinking the influence of less useful variables. One single model (ANN/LASSo) was developed to predict 5-year disease-specific survival following surgery for invasive IPMN [25]. They retrospectively analysed data from 440 patients who underwent surgical resection for IPMN and used clinical and pathological data including age, gender, tumour size and location, surgical details, histological grade, treatment history, and TNM stage. The authors showed the feasibility of ANN application with the use of LASSO in predicting survival post-surgery for invasive IPMN; however, the model performances were comparable to traditional logistic regression, with no statistically significant difference. Few other studies (n = 5), have utilised LASSO as part of their development of models, proposing that LASSO regression could select the most effective feature subset and achieve a better performance [20,25,28,42,59]. While AI applications for diagnosis are increasing, its use in prognostic modelling remains limited. Given the high morbidity and mortality associated with pancreatic surgery, developing predictive models for postoperative outcomes is an essential avenue for future investigation. Such tools could enhance patient counselling, risk stratification, and follow-up strategies.

### 4.2. Limitations

A recurring limitation across many studies was the relatively small sample size, particularly in those focused on differentiating between specific pancreatic cyst types such as IPMN, MCN, and SCN. While some models, especially those aimed at lesion detection or segmentation, were developed using larger datasets, the majority were retrospective in nature. For AI applications to be clinically robust, prospective, multicentre studies with diverse patient populations will be essential to ensure external validity. Moreover, there was notable heterogeneity across studies in both the input data used and the outcome definitions applied. Some models were built exclusively on imaging features, while others integrated additional texture features, clinical variables, or molecular data. It remains unclear whether incorporating texture features such as calcification, central scarring, and other density differences consistently improves model performance. For instance, Chen et al. demonstrated that a logistic regression model combining radiological and texture features achieved superior diagnostic performance (AUC 0.932) compared to a model based solely on imaging features (AUC 0.84) [20]. In contrast, Awe et al. developed an ensemble model that showed no significant difference between the two approaches, with AUCs of 0.73 (radiological plus clinical parameters) and 0.72 (radiological parameters alone) [60]. This inconsistency may be attributed to differences in sample size, model architecture, or feature engineering methodologies. Future studies should further investigate the role of texture features by applying multiple machine learning models and validating findings using external datasets. Several studies included in our review incorporated radiomics as one of multiple parameters in model development. However, a key limitation in radiomics-based approaches is the lack of image standardisation, which can introduce additional heterogeneity into model performance and reproducibility. This variability arises from differences in acquisition protocols, scanner types, segmentation methods, and the software tools used for feature extraction and analysis [61]. Rather than replacing clinical judgement, these studies suggest that machine learning models should be integrated into existing decision-making processes to enhance diagnostic precision and improve patient outcomes.

### 4.3. Future Directions

This review highlights several important directions for future research aimed at translating AI-based tools for pancreatic cyst management into clinical practice. First, there is a critical need for robust external validation of AI models. Most studies reviewed were retrospective and lacked independent validation cohorts, which limits generalisability. Future studies should prioritise prospective, multicentre research designs and ensure transparent reporting in line with established frameworks such as TRIPOD and PROBAST. This will enhance reproducibility, facilitate model comparison, and build clinical trust. Secondly, addressing the issue of image and radiomics standardisation is essential. The current heterogeneity in imaging acquisition protocols, segmentation techniques, and radiomic feature extraction pipelines poses a major barrier to reproducibility. Standardising these protocols across institutions will be vital for building generalisable AI tools. International collaborations may play a crucial role in this effort, enabling the creation of large, diverse, and standardised imaging datasets. Thirdly, the future development of AI models should go beyond diagnostic accuracy and focus on demonstrating clinical utility. Studies should assess whether the use of AI leads to measurable improvements in patient outcomes, such as reduced rates of unnecessary surgery, earlier identification of high-risk lesions, or improved long-term survival. Incorporating tools such as decision curve analysis, impact studies, and cost-effectiveness models will help determine the practical value of AI in real-world settings. Finally, the development of explainable and interpretable AI models remains a priority. Many deep learning-based approaches, despite their strong performance, operate as “black boxes”, which limits clinical adoption. Future work should incorporate explainability frameworks such as attention maps or feature importance metrics to ensure that model predictions are transparent and clinically meaningful. Integrating such interpretable tools into multidisciplinary workflows can enhance clinical decision-making, support individualised patient care, and promote clinician confidence in AI-assisted strategies.

## 5. Conclusions

This systematic review identified 31 studies evaluating the application of artificial intelligence in the diagnosis and management of pancreatic cystic lesions. Overall, the included models demonstrated higher median AUC values and accuracies compared to existing clinical guidelines. The risk of bias across studies was generally high, underscoring the need for future research to develop and validate models in accordance with established reporting and methodological standards, such as TRIPOD and PROBAST.

## Figures and Tables

**Table 1:** Summary of high quality and externally validated AI studies for pancreatic cyst management/diagnosis.

Author (Year)	AI Model	Sample Size	Parameters	Clinical Focus	Performance	Compared to Guidelines/Clinicians	Limitations
Wang et al. (2022) [26]	Ensemble	363	CT images	Benign vs. malignant PCLs	AUC = 0.91, Acc = 0.84, Sens = 0.96, Spec = 0.68	Performance similar to senior radiologist, but better than juniors	Retrospective
Deng et al. (2024) [33]	LR	388	CT images and clinical	Benign vs. malignant PCLs	AUC = 0.95, Acc = 0.90, Sens = 0.96, Spec = 0.83	Performance better than ACG and European guidelines	Retrospective
Watson et al. (2021) [34]	CNN	27	CT images	Benign vs. malignant PCLs	Acc = 0.89	Performance better than Fukuoka guideline, reducing unnecessary surgeries	Small sample size; no AUC data; retrospective
Schulz et al. (2022) [38]	CNN	70	EUS images	IPMN grading	Acc = 0.99, Sens = 1, Spec = 0.99	Outperformed existing guidelines	Small prospective cohort (7/70)
Cui et al. (2021) [42]	LR (LASSO)	202	CT/MRI radiomics	BD-IPMN grading	AUC = 0.88, Sens = 0.90, Spec = 0.79	Not specified	Retrospective; moderate sample size
Oh et al. (2021) [43]	CNN	111	EUS images	Segmentation of PCLs	Acc = 0.97, Sens = 0.72, Spec = 0.99	Comparable to human readers/interpretation	Lower sensitivity; requires manual segmentation
Park et al. (2022) [30]	CNN	2044	CT (noncontrast)	Cystic vs. solid lesions	AUC = 0.87–0.91, Acc = 0.83–0.86	Comparable to radiologists if the lesion size is 1.0 cm or higher	Retrospective; performance varies by lesion size
Springer et al. (2019) [24]	Supervised	862	CT, MRI, EUS images	Management decision support	Acc = 0.69, Sens = 0.91, Spec = 0.54	Higher accuracy compared to local standard of care accuracy	Retrospective

Abbreviations: ACG (American College of Gastroenterology); AUC (area under the receiver operating characteristic curve); Acc (accuracy); BD-IPMN (branch-duct intraductal papillary mucinous neoplasm); CNN (convolutional neural network); CT (Computed Tomography); EUS (endoscopic ultrasound); IPMN (intraductal papillary mucinous neoplasm); LASSO (least absolute shrinkage and selection operator); LR (logistic regression); MRI (Magnetic Resonance Imaging); PCL (pancreatic cystic lesion); Sens (sensitivity); Sepc (specificity).

## Data Availability

No new data were created or analysed in this study. Data sharing is not applicable to this article.

## Appendix A

**Table A1:** Studies on pancreatic cystic lesion malignancy prediction and their characteristics.

Author	Category	Type	Data Source	AI Model	Country	Imaging Method	Number of Patients	Parameter Used	Endpoint	Performance	Conclusion
Wang et al. (2022) [26]	Preoperative diagnosis	Retrospective	Multicentre	Ensemble	China	CT	n = 363	Radiological	Differentiating benign vs. malignant cysts	AUC = 0.91, Accuracy = 0.84, Sensitivity = 0.96, Specificity = 0.68	Deep learning model can differentiate benign and malignant PCLs with stable diagnostic performance. Performance similar to senior radiologist, but better than junior radiologists and surgeons.
Deng et al. (2024) [33]	Preoperative diagnosis	Retrospective	Multicentre	Logistic Regression	China	CT	n = 388	Radiological and Clinical (sex, age, jaundice, pancreatitis, CEA and CA19-9 levels)	Differentiating benign vs. malignant cysts	AUC = 0.948, Accuracy = 0.900, Sensitivity = 0.963, Specificity = 0.826	The model can accurately identify malignant PCLs in patients with worrisome or high-risk features. The diagnostic performance is better than the European guidelines and the ACG guidelines.
Saraiva et al. (2024) [62]	Preoperative Diagnosis	Retrospective	Multicentre	CNN	Portugal, USA	EUS	n = 378, EUS images = 126,000	Endoscopic EUS images	Classification of PCLs and PSL	Accuracy = 99%, Sensitivity = 98.9%, Specificity = 99.1%,	CNN capable of detection and differentiation of PCN (namely between M-PCN and NM-PCN) and PSL (namely between P-DAC and P-NET).
Watson et al. (2021) [34]	Preoperative Diagnosis	Retrospective	Single centre	CNN	USA	CT	n = 27	Radiological	Differentiating benign vs. malignant cysts	Accuracy = 88.9%, AUC = N/A	CNN model can improve diagnostic precision and reduce unnecessary surgeries. Better performance compared to Fukuoka guideline

Abbreviations: ACG (American College of Gastroenterology); AUC (area under the receiver operating characteristic curve); CA19-9 (carbohydrate antigen 19-9); CEA (carcinoembryonic antigen); CNN (convolutional neural network); CT (Computed Tomography); EUS (endoscopic ultrasound); M-PCN (malignant pancreatic cystic neoplasm); N/A (Not Available); NM-PCN (non-malignant pancreatic cystic neoplasm); PCL (pancreatic cystic lesion); PCN (pancreatic cystic neoplasm); PDAC (pancreatic ductal adenocarcinoma); P-NET (pancreatic neuroendocrine tumour); PSL (pancreatic solid lesion).

**Table A2:** Studies on classification of pancreatic cysts subtypes and their characteristics.

Author	Category	Type	Data Source	AI Model	Country	Imaging Method	Number of Patients	Parameter Used	Endpoint	Performance	Conclusion
Awe et al., 2021 [60]	Preoperative diagnosis	Retrospective	Single centre	Ensemble	USA	CT	n = 99	Radiological, clinical, and radiomics	To differentiate mucinous from non-mucinous pancreatic cysts	AUC = 0.73 Accuracy = 0.74 Sensitivity = 0.77 Specificity = 0.61	ML principles can be applied to radiomics data of pancreatic cysts to help detect mucinous phenotypes, but the integration of radiologic and clinical features with texture feature radiomics data does not improve the performance of the mucinous classifier.
Liang et al., 2022 [63]	Preoperative diagnosis	Retrospective	Single centre	Logistic regression	China	CT	n = 193	Radiological	To differentiate between SCN, MCN, and IPMN	AUC = 0.973 Accuracy = 0.92 Sensitivity = 0.86 Specificity = 1	Radiomics-based models using CT data can be used to classify pancreatic cystic tumours. Specific morphological features like tumour location, number of cysts, and wall calcification can improve the classification of pancreatic cystic lesions.
Zhang et al., 2022 [64]	Preoperative diagnosis	Retrospective	Single centre	CNN/GNN	China	CT	n = 263	Radiological	To differentiate between SCN, MCN, SPN, and IPMN	AUC = 0.856 Accuracy = 0.74	Model can effectively classify PCNs into benign and malignant types, as well as provide specific classifications for the different PCN subtypes, with high accuracy even on small and imbalanced datasets without requiring exact segmentation of the neoplasm.
Chu et al., 2022 [27]	Preoperative diagnosis	Retrospective	Single centre	Random forest	USA	CT	n = 214	Radiomics	To classify cysts: IPMNs, MCNs, SCAs, SPNs, and cystic PNETs	AUC = 0.94 Accuracy = 0.94 Sensitivity = 0.94 Specificity = 0.93	Radiomics-based machine learning approach can achieve equivalent performance as an experienced academic radiologist in classifying different types of pancreatic cystic neoplasms. No statistical significance due to small sample size
Tian et al., 2024 [19]	Preoperative diagnosis	Retrospective	Single centre	CNN	China	MRI	n = 314	Radiomics and clinical	To differentiate between SCN and MCN	AUC = 0.971 Accuracy = 0.92 Specificity = 0.93	The proposed CBAM DenseNet model incorporating clinical features achieved strong performance in classifying pancreatic cystic tumours (SCN and MCN.
Chen et al., 2021 [20]	Preoperative diagnosis	Retrospective	Multicentre	Logistic regression	China	CT contrast	n = 128	Radiological and clinical	To differentiate between SCNs and MCNs	AUC = 0.88 Sensitivity = 0.99 Specificity = 0.84	Logistic regression model combining radiological features and CT texture features is more effective in distinguishing pancreatic SCNs from MCNs compared to a model using only radiological features.
Vilas-Boas et al., 2022 [65]	Preoperative diagnosis	Prospective	Single centre	CNN	Portugal	EUS	n = 5505	Radiological (EUS image)	To differentiate MCN from non-MCNs	AUC = 1.0 Accuracy = 0.99 Sensitivity = 0.98 Specificity = 0.99	The model significantly outperforms EUS alone, which has a variable accuracy of 48–94%. The deep learning approach can aid risk stratification and clinical decision-making for pancreatic cystic lesions.
Wei et al., 2019 [28]	Preoperative diagnosis	Retrospective	Single centre	SVM	China	CT	n = 214	Radiomics and clinical	To differentiate SCN from other cystic neoplasms	AUC = 0.77 Sensitivity = 0.69 Specificity = 0.71	The model can provide a powerful reference for the diagnosis. LASSO regression could select the most effective feature subset and achieve a better performance. Clinician diagnosis was 37.3% from retrospective study, suggesting models can support the diagnosis.
Yang et al., 2019 [59]	Preoperative diagnosis	Retrospective	Single centre	Random forest	China	MRI	n = 314	Radiomics	To differentiate SCN and MCN	AUC = 0.75 Accuracy = 0.83 Sensitivity = 0.85 Specificity = 0.83	Demonstrated that CT textural features can differentiate between SCAs and MCAs with reasonable accuracy.

Abbreviations: AUC (area under the receiver operating characteristic curve); BMI (Body Mass Index); CAD (Computer-Aided Diagnosis); CBAM (Convolutional Block Attention Module); CNN (Convolutional Neural Network); CT (Computed Tomography); EUS (endoscopic ultrasound); GNN (Graph Neural Network); IPMN (intraductal papillary mucinous neoplasm); LASSO (least absolute shrinkage and selection operator); MCN (mucinous cystic neoplasm); MRI (Magnetic Resonance Imaging); MPD (main pancreatic duct); PCN (pancreatic cystic neoplasm); PCL (pancreatic cystic lesion); PNET (pancreatic neuroendocrine tumour); SCN (serous cystic neoplasm); SPN (solid pseudopapillary neoplasm); SVM (support vector machine).

**Table A3:** Studies on the classification of IPMN into its subtypes.

Author	Category	Type	Data Source	AI Model	Country	Imaging Method	Number of Patients	Parameter Used	Endpoint	Performance	Conclusion
Hernandez-Barco et al., 2023 [35]	Preoperative diagnosis	Prospective	Single centre	Linear support vector machine (SVM)	USA	-	n = 575	Clinical	To classify IPMN	AUC = 0.82 Accuracy = 77 Sensitivity = 83 Specificity = 72	Linear SVM-based machine learning model can be useful to better determine which patients diagnosed with an IPMN might benefit from observation versus surgical resection. Better performance compared to Fukuoka and AGA guideline with better sensitivity and similar specificity
Kiritani et al., 2023 [36]	Preoperative diagnosis	Prospective	Multicentre	SVM	Japan, Helsinki	EUS/ERCP	n = 49	863 peak intensities obtained from the PESI-MS analysis	To classify IPMN	AUC = 0.924 Sensitivity = 0.88 Specificity = 0.88 Accuracy = 0.88	The combination of PESI-MS and machine learning (SVM) was able to accurately distinguish advanced IPMN from low-grade IPMN using 130 key variables. Accuracy outperforms current guideline; Fukuoka
Salanitri et al., 2022 [66]	Preoperative diagnosis	Retrospective	Multicentre	Vision transformers (neural network)	USA, Italy	MRI	n = 139	Imagistic parameter	To classify IPMN	Accuracy = 0.70 Precision = 0.67 Recall = 0.64	The transformer-based model achieved promising results that can be used for routine IPMN risk stratification. Training the transformer model was easier than training conventional CNN models, and it also generalised better
Machicado et al., 2021 [37]	Preoperative diagnosis	Prospective	Single centre	CNNs	USA	EUS-nCLE	n = 35	Histology	To risk stratify IPMN	Accuracy = 0.86 Sensitivity = 0.83 Specificity = 0.88	EUS-nCLE-based CNN-CAD algorithms can accurately risk stratify IPMNs and outperform current clinical guidelines (Fukuoka, AGA) in diagnosing advanced neoplasia (high-grade dysplasia/adenocarcinoma) in IPMNs
Dominik Schulz et al., 2022 [38]	Preoperative diagnosis	Retrospective/Prospective (7 patients for testing recruited prospectively)	Single centre	CNN	Germany	EUS	n = 70	EUS images	To classify IPMN	Accuracy = 0.99 Sensitivity = 1 Specificity = 0.97	Deep learning model can accurately predict the histological grading of IPMNs from endoscopic ultrasound (EUS) images, with significantly higher accuracy compared to existing clinical guidelines.
Sijia Cui et al., 2021 [42]	Preoperative diagnosis	Retrospective	Multicentre; China 3 hospitals	Logistic regression (LASSO-based feature selection)	China	MRI and CET images	n = 202	Radiomics	To classify BD-IPMN	AUC = 0.884 Sensitivity = 0.9 Specificity = 0.79	Preoperative pathological grade of BD-IPMNs could be predicted effectively using the developed nomogram model combining the radiomic signature and tumour clinical characteristics.
Jae Seung Kang et al., 2020 [39]	Preoperative diagnosis	Retrospective cohort study	Multicentre; international	AutoML package	Korea	CT, MRI, EUS	n = 3708	Clinical and radiological	Differentiation between benign and malignant IPMNs	AUC = 0.73	Both ML and LR models showed comparable predictive performance. Logistic regression was considered more practical for clinical use due to simplicity and ease of interpretation.
Takamichi Kuwahara et al., 2019 [29]	Preoperative diagnosis	Retrospective	Single centre	CNN	Japan	EUS	50 patients	EUS images and clinical	Prediction of malignancy in IPMNs	AUC = 0.98 Sensitivity = 0.95 Specificity = 0.92 Accuracy = 0.94	AI via deep learning algorithm may be a more accurate and objective method to diagnose malignancies of IPMNs in comparison to human diagnosis and conventional EUS features. The model showed better performance compared to human diagnosis and traditional logistic regression model.

Abbreviations: AGA (American Gastroenterological Association); AUC (area under the receiver operating characteristic curve); BD-IPMN (branch-duct intraductal papillary mucinous neoplasm); CA19-9 (carbohydrate antigen 19-9); CEA (carcinoembryonic antigen); CNN (convolutional neural network); CT (Computed Tomography); DL (deep learning); EUS (endoscopic ultrasound); ERCP (Endoscopic Retrograde Cholangiopancreatography); GBM (Gradient Boosting Machine); GLM (Generalised Linear Model); IPMN (intraductal papillary mucinous neoplasm); LASSO (least absolute shrinkage and selection operator); LR (logistic regression); MPD (main pancreatic duct); MRI (Magnetic Resonance Imaging); nCLE (needle-based confocal laser endomicroscopy); PESI-MS (probe electrospray ionisation mass spectrometry); SVM (support vector machine); T1-w (T1-weighted Imaging); T2-w (T2-weighted Imaging); XG Boost (Extreme Gradient Boosting).

**Table A4:** Studies on pancreatic cysts segmentation and identification.

Title	Category	Type	Data Source	AI Model	Country	Imaging Method	Number of Patients	Parameters Used	Endpoint	Performance	Conclusion
Oh et al., 2021 [43]	Preoperative diagnosis	Retrospective	Single centre	CNNs	Korea	EUS	n = 111	Manual segmentation	Automatic segmentation of pancreatic cyst lesions (PCLs) on endoscopic ultrasonography (EUS) images	Accuracy = 0.972 Specificity = 0.989 Sensitivity = 0.723	The deep learning-based Attention U-Net model performed best for PCL segmentation on the internal test data compared to other models.
Park et al., 2022 [30]	Preoperative diagnosis	Retrospective	Multicentre	CNNs	Korea	CT contrast	n = 2044	Manual segmentation	To identify patients with various solid and cystic pancreatic neoplasms	AUC = 0.87 Sensitivity = 83.3 Specificity = 82.7 Accuracy = 82.9	Performances were comparable to radiologists if the lesion size is 1.0cm or higher.
Abi Nader et al., 2023 [67]	Preoperative diagnosis	Retrospective	Europe, USA, and Brazil	CNNs	France	CT	n = 2890	Radiological	To detect the presence of pancreatic lesions and identify main pancreatic duct dilatation with high accuracy	IPMN AUC = 0.98 Sensitivity = 0.94 Specificity = 0.95 MPD AUC = 0.97 Sensitivity = 0.94 Specificity = 0.90	Effectively detect pancreatic neoplasms and identify cases with MPD dilatation.
Kooragayala et al., 2022 [68]	Preoperative diagnosis	Retrospective	Single centre	Natural language processing (NLP)	USA	CT	n = 18,769	Radiological	Identification of potentially concerning pancreatic lesions	Sensitivity = 0.33 Specificity = 0.99 PPV = 0.25 NPV = 0.99	NLP technology could be clinically useful to more efficiently relay important incidental findings from CT imaging to patients and providers
Konikoff et al., 2024 [69]	Preoperative Diagnosis	Retrospective	Single centre	CNN	Israel	EUS	n = 1497	EUS images	Real-time AI-based detection and segmentation of pancreatic lesions on EUS	Accuracy = 0.93 AUC = 0.89 Sensitivity = 0.48 Specificity = 0.98	Improved lesion detection
Duh et al., 2023 [31]	Preoperative Diagnosis	Retrospective	Single Centre	CNN	Spain	CT	n = 335	Manual segmentation	Automated detection of pancreatic cystic lesions (PCLs) on CT scans	Sensitivity = 0.93 Specificity = 0.82	AI outperforms traditional radiological assessment in early detection of pancreatic cysts
Abel et al., 2021 [32]	Preoperative diagnosis	Retrospective	Single centre	CNN	Switzerland	CT	n = 543	Radiological	Detection of pancreatic cystic lesions using deep learning	Sensitivity = 0.87	AI can assist radiologists in pancreatic cyst detection.

Abbreviations: AUC (area under the receiver operating characteristic curve); CAD (Computer-Aided Detection); CADe (Computer-Aided Detection System); CNN (convolutional neural network); CT (Computed Tomography); EUS (endoscopic ultrasound); IPMN (intraductal papillary mucinous neoplasm); MCN (mucinous cystic neoplasm); MPD (main pancreatic duct); NET (neuroendocrine tumour); NLP (natural language processing); nnU-Net (No-New-Net (self-configuring deep learning framework)); NPV (Negative Predictive Value); PDAC (pancreatic ductal adenocarcinoma); PCL (pancreatic cystic lesion); PPV (Positive Predictive Value); ViT (vision transformer).

**Table A5:** Studies on management of pancreatic cysts and prediction of prognosis post pancreatic cyst surgery.

Title	Category	Type	Data Source	AI Model	Country	Imaging Method	Number of Patients	Parameters Used	Endpoint	Performance	Conclusion
Ferres et al., 2024 [23]	Preoperative diagnosis	Retrospective	USA, Europe and Asia	Ensemble	USA	-	n = 850	Clinical	Stratification into surgery, surveillance, or discharge	Discharge = 0.93 Surveillance = 0.84 Surgery = 0.83	The EBM model reduced unnecessary surgeries by 59% and increased correct surgeries by 7.5% compared to clinical care. The model provided interpretable explanations, showing which features contributed most to a decision
Springer et al., 2019 [24]	Preoperative diagnosis	Retrospective	USA, Europe, and Asia	Supervised model	USA	CT, MRI, EUS	n = 862	Molecular, clinical, and radiological	Management of pancreatic cysts	Sensitivity = 0.9 Specificity = 0.54	Comp cyst cannot replace the conventional clinical tools, instead it would contribute towards making informed diagnosis, as it has a higher accuracy compared to current standard of care accuracy (56%)
Aronsson et al., 2021 [25]	Prognosis evaluation	Retrospective	USA	ANN, LASSO	Sweden	-	n = 440	Clinical	Prediction of 5-year disease-specific survival (DSS) after surgical treatment	ANN model Accuracy = 0.81 Precision = 0.85 Specificity = 0.52 Lasso Accuracy = 0.80 Precision = 0.85 Specificity = 0.52	ANN and LASSO models were able to accurately predict 5-year disease-specific survival after surgery for invasive IPMN, with performance comparable to traditional logistic regression

Abbreviations: AJCC (American Joint Committee on Cancer); ANN (artificial neural network); CEA (carcinoembryonic antigen); CFMM (Cyst Fluid Molecular Markers); CT (Computed Tomography); DSS (disease-specific survival); EBM (Explainable Boosting Machine); EUS (endoscopic ultrasound); IPMN (intraductal papillary mucinous neoplasm); LASSO (least absolute shrinkage and selection operator); MCN (mucinous cystic neoplasm); MRI (Magnetic Resonance Imaging); VEGF-A (Vascular Endothelial Growth Factor A); VHL (Von Hippel-Lindau).
